# Fabrication of *In Situ* Grown Hydroxyapatite Nanoparticles Modified Porous Polyetheretherketone Matrix Composites to Promote Osteointegration and Enhance Bone Repair

**DOI:** 10.3389/fbioe.2022.831288

**Published:** 2022-02-28

**Authors:** Ningning Wang, Desheng Qi, Lu Liu, Yanlin Zhu, Hong Liu, Song Zhu

**Affiliations:** ^1^ Department of Prosthetic Dentistry, School and Hospital of Stomatology, Jilin University, Changchun, China; ^2^ College of Chemistry, Engineering Research Center of Special Engineering Plastics, Ministry of Education, Jilin University, Changchun, China; ^3^ Department of Stomatology, China-Japan Friendship Hospital, Jilin University, Changchun, China; ^4^ Department of Oral Implantology, School and Hospital of Stomatology, Jilin University, Changchun, China; ^5^ Department of General Dentistry, School and Hospital of Stomatology, Jilin University, Changchun, China

**Keywords:** nanohydroxyapatite, polyetheretherketone, differentiation, animal model, osteointegration

## Abstract

The repairment of critical-sized bone defects is a serious problem that stimulates the development of new biomaterials. In this study, nanohydroxyapatite (nHA)-doped porous polyetheretherketone (pPEEK) were successfully fabricated by the thermally induced phase separation method and hydrothermal treatment. Structural analysis was performed by X-ray diffraction. The water contact angles and scanning electron microscopy were measured to assess physical properties of surfaces. The mechanical strength of the composites is also determined. Microcomputed tomography is used to characterize the nHA content of the composites. The *in vitro* bioactivity of the composites with or without nHA was investigated by using murine pre-osteoblasts MC3T3-E1, and the results of cytotoxicity and cell proliferation assays revealed that the cytocompatibility of all specimens was good. Adherence assays were employed to examine the adhesion and morphology of cells on different materials. However, nHA-doped composites induced cell attachment and cell spreading more significantly. Osteogenic differentiation was investigated using alkaline phosphatase activity and alizarin red staining, and these *in vitro* results demonstrated that composites containing nHA particles enhanced osteoblast differentiation. Its effectiveness for promoting osteogenesis was also confirmed in an *in vivo* animal experiment using a tibial defective rat model. After 8 weeks of implantation, compared to the pure PEEK and pPEEK without nHA groups, the nHA-pPEEK group showed better osteogenic activity. The results indicate that the nHA-pPEEK composites are possibly a well-designed bone substitute for critical-sized bone defects by promoting bone regeneration and osteointegration successfully.

## 1 Introduction

Bone defects, which are caused by trauma, tumor surgery, and inflammation, affect the quality of life of patients seriously and remain a major clinical challenge (Huang et al., 2019; Xie et al., 2020). Autologous bone grafts are considered the gold standard as bone implants due to their ideal osteo-inductive properties, preventing the histocompatibility problem. However, the associated risk of donor site disease and limited supplementation are disadvantages of the treatment (Beigi et al., 2019; Kohli et al., 2021). Metals (e.g., titanium alloys) are widely used as prostheses because of the high mechanical strength and excellent frictional resistance (Yan et al., 2018). Some disadvantages, however, have been pointed out for metal implants: image distortion in postoperative observations, stress shielding effect on the bone around the implant, and the need of additional surgery for removal (Hughes et al., 2018; Ko et al., 2021).

In recent years, there has been rising interest to develop the new medical devices based on polyetheretherketone (PEEK) because of its good biocompatibility and mechanical properties that can match the cortical bone tissue (Vaezi et al., 2016; Feng et al., 2019). Unlike metal implants, which create artifacts in radiography, computed tomography (CT), and magnetic resonance imaging (MRI), PEEK does not generate such artifacts (Fukuda et al., 2018; Sunarso et al., 2020). However, PEEK is not a material with bioactivity, which possesses poor osseointegration, owing to its bioinert nature (Dai et al., 2019). Therefore, the development of new modification techniques to enhance the bioactivity of PEEK is an important requirement.

Several strategies have been studied to improve the bioinert nature of PEEK with bioactive materials (Wan et al., 2020), and synthetic hydroxyapatite (HA), which is most similar to mature bone, has been widely applied as a bone substitute for the requirement of bone defects in orthopedic surgery (Pujari-Palmer et al., 2017; Zakrzewski et al., 2020). To enhance the osteointegration of PEEK-based implants, several techniques have already been developed for various orthopedic uses: 1) the incorporation of HA into the PEEK matrix, 2) coating PEEK implants with HA, and 3) adding porosity into PEEK implants (Zhong et al., 2019). Due to the natural structure of native bone tissues, pores in implants play a key role in bone regeneration (Lin et al., 2019) because biocompatible porous implants that mimic the natural bone structure have given the most hopeful results due to their promoting effect on cell proliferation and neovascularization (Paraš et al., 2020). Among these methods, the use of HA combined with the porosity has been considered a desirable technology for improving the osteointegration of PEEK-based devices (Torstrick et al., 2018). Some investigators have reported that the incorporation of bioactive molecules and porosity to PEEK are effective approaches to improve the osteointegration of PEEK-based implants (Chubrik et al., 2020; Conrad and Roeder, 2020; Swaminathan et al., 2020; Huang et al., 2021; Venkatraman et al., 2021).

In summary, two major methods have been performed to improve the biological activity of porous PEEK (po-PEEK) materials with the synthetic HA. One approach is to fabricate PEEK-based composites by incorporating bioactive molecules into PEEK substrates, but this may influence the mechanical strength. Another strategy is to activate the surface of PEEK with HA coating. However, PEEK surface coatings may lead to insufficient cohesion and delamination, resulting in tissue inflammation (Han et al., 2019).

**SCHEME 1:**
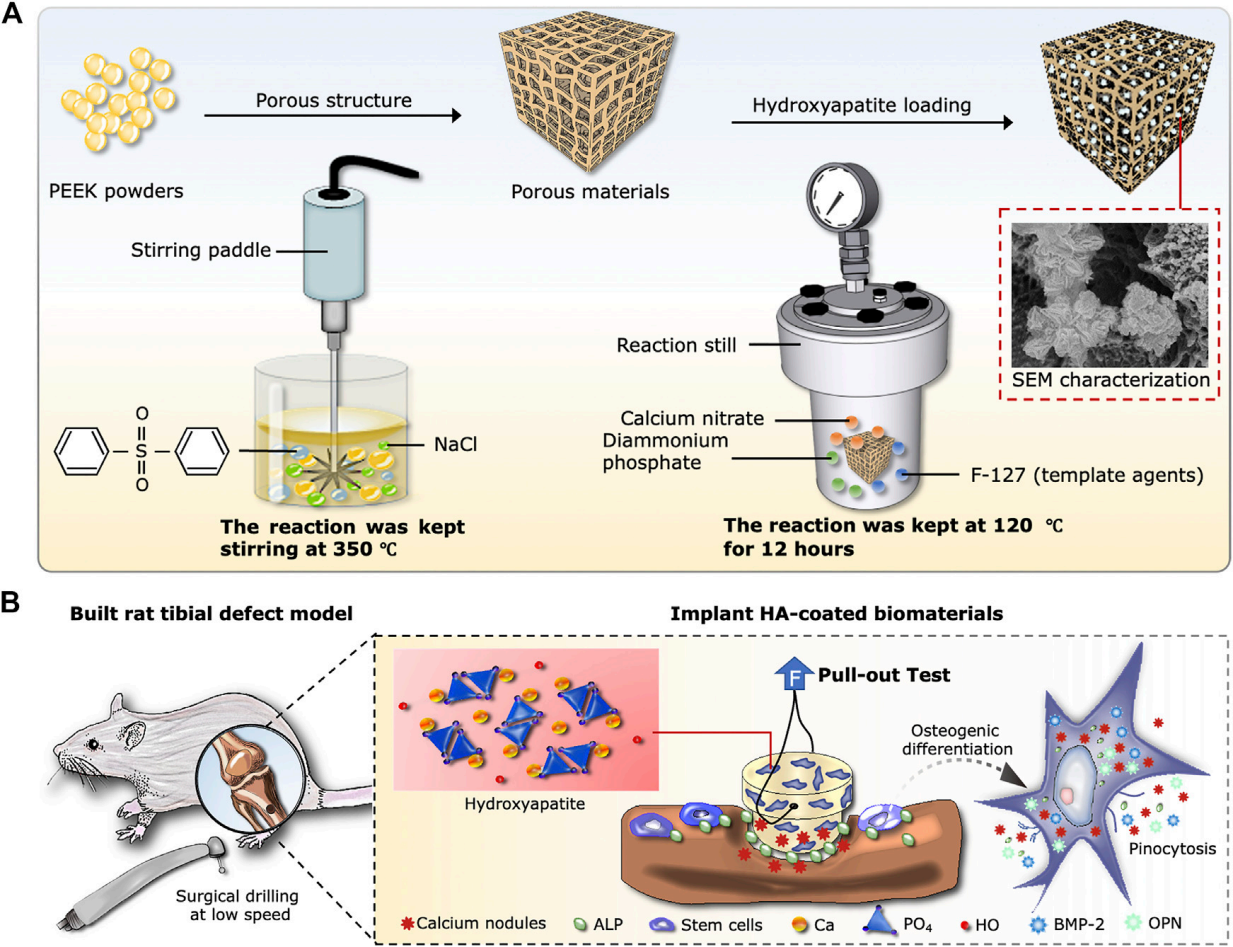
Design of the experiment. **(A)** Schematic diagram of synthesis process, including the Po-PEEK with thermally induced phase separation and nHA with hydrothermal methods. **(B)** Implantation procedure of rat tibial defect model and cellular responses around the host bones after the operation.

The objective in this study is to prepare a new kind of porous composite based on porogens in a PEEK matrix by using a novel experimental technique. Subsequently, HA is generated *in situ* inside the porosity of Po-PEEK biomaterial *via* hydrothermal synthesis, aiming to develop a bioactive composite with good biological performances for bone regeneration and reconstruction. A schematic diagram of the experimental protocol is illustrated in Scheme 1.

## 2 Materials and Methods

### 2.1 Preparation of Porous PEEK

#### 2.1.1 Fabrication

Briefly, diphenyl sulfone was added into a 1 L round-bottom flask and heated to 140°C until completely dissolved. PEEK powder (melt flow rate = 13, offered by Jilin University Special Plastic Engineering Research Center) was added and slowly warmed to 350°C. Then, the solution was stirred until the powder was dissolved completely. Finally, sodium chloride was added, and the solution continued to be stirred until it was homogeneous. The product was poured into the mold and cooled to room temperature. Subsequently, the resulted polymer was further purified by Soxhlet extractions for 3 days with acetone and distilled water to remove diphenyl sulfone and excess inorganic salts. Throughout the manuscript, two types of Po-PEEK with different solid contents were synthesized, and different degrees of compression were processed to improve the mechanical properties. Materials were divided into four groups: PK50-20% (50% solid content and 20% compression), PK50-10% (50% solid content and 10% compression), PK50 (50% solid content), and PK40 (40% solid content). The solid content was calculated as shown below:

$$Solid\;content=\;\frac{M_{PEEK}}{M_{PEEK}+M_{diphenyl\;sulfone}}$$

Where M_PEEK_ and M_diphenyl_ _sulfone_ represented the mass of PEEK powder and diphenyl sulfone in the reaction system, respectively.

#### 2.1.2 SEM Observation

The material was broken up with liquid nitrogen, and the cross-sections were sputtered with gold for 60 s. The samples were then observed and photographed with a scanning electron microscope (SEM, Hitachi S4800; Japan).

#### 2.1.3 Density and Porosity

The density of materials was tested by a density/specific gravity meter DA-510 (Kyoto Electronics Manufacturing, Kyoto, Japan) at room temperature and the porosity was calculated based on the formula as follows:

$$P=1-\;\frac{\rho }{\rho_{0}}$$

where P denoted the porosity of fabricated material, and ρ indicated the density of the material in its natural state, and ρ_0_ represented the absolute solid density of this material.

#### 2.1.4 Mechanical Strength

The materials were cut into 1 cm cubes for tests of mechanical properties, with an Instron universal tester 5,966 (Instron, Norwood, MA, United States).

### 2.2 Fabrication of Nanohydroxyapatite-Doped Porous PEEK

#### 2.2.1 Synthesis Process

The nanohydroxyapatite (nHA) was synthesized in the laboratory *via* a hydrothermal treatment method. Specifically, di-ammonium hydrogen phosphate (AR grade) was dissolved in 10 ml of Millipore water, and Po-PEEK with 50% solid content was immersed in it under vacuum to make the solution infiltrate the pores of the samples. Afterwards, the materials were frozen at −20°C and lyophilized. Calcium nitrate and pluronic F127 were dissolved in 10 ml of Millipore water and stirred until complete homogenization. Pluronic F127 was selected as a soft template to obtain the porous structure (Gong J. et al., 2020). The lyophilized samples were immersed in the solution of calcium nitrate under vacuum to make theoluteion infiltrate the pores again, and the precipitation was carried out by adding di-ammonium hydrogen phosphate dropwise to the calcium nitrate solution under constant stirring, followed by being transferred into reaction kettles at 120°C for about 12 h. Following the termination of the experiment, all samples were washed with distilled water to remove remaining salts and Pluronic F127, keeping the samples dried at 60°C in the vacuum oven.

#### 2.2.2 SEM Observation

The operation was performed essentially as described previously.

#### 2.2.3 Water Contact Angle Test

The water contact angle of the sample surface was analyzed by the contact angle-measuring instrument (DSA25, Kruss, Germany). The test materials were then cut into samples of 20 mm length, 10 mm width, and an average thickness of 2 mm. The droplets were laid onto the sample surfaces, and the images were captured using the camera. The contact angle was measured by recording the contact angle values.

#### 2.2.4 X-Ray Diffraction Analysis (XRD)

The synthesized nHA powders on an XRD grid were exposed to the measurements, and the structures of which were characterized by using an X-ray diffract meter (XRD-6000; Shimadzu, Tokyo, Japan) in the 2θ range from 10° to 80° at a scanning rate of 10°/min.

#### 2.2.5 Experiment Sizes of Samples

**FIGURE 1:**
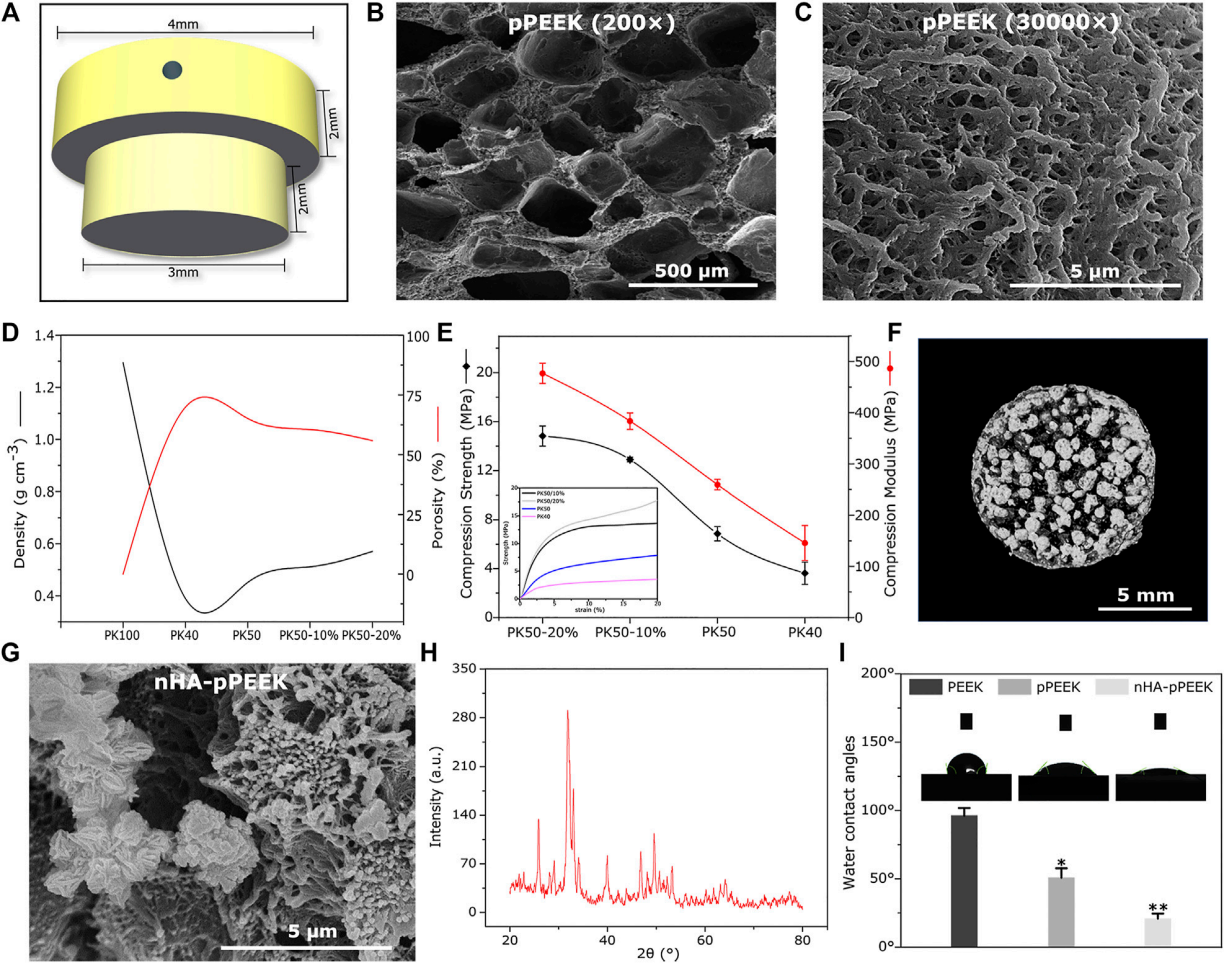
Characterization. **(A)** 3D schematic of the implant material **(B,C)** SEM images of porous materials with different magnification. **(D)** Density and porosity of all groups with different solid content and compressive degrees. **(E)** Mechanical strength of all groups with different solid content and compressive degrees. **(F)** Micro-CT image of the material in the PK50-20% group. **(G)** SEM picture of synthetic nHA. **(H)** XRD diffractogram for samples of synthetic nHA. **(I)** The water contact angle analysis of the different samples.

The experiments were conducted using two different sizes of materials. For *in vivo* experiments, the samples were 1 mm thick and 10 mm in diameter. *In vivo* samples were T-shaped columns, with 3 mm diameter at the bottom and 4 mm diameter at the top. Both sections measured 2 mm in length for a total length of 4 mm. The top section had a 0.75 mm diameter transverse hole for pull-out testing. Figure 1A shows a schematic of this appearance.

### 2.3 *In Vitro* Assays

#### 2.3.1 Cytotoxicity Assay

*In vitro* cytotoxicity was measured by MTT assays with MC3T3-E1 osteoblasts, which were purchased from the American Type Culture Collection (Manassas, VA, United States). The cells were grown in DMEM with 10% fetal bovine serum (FBS) and 1% penicillin–streptomycin (all Sigma-Aldrich, MO, United States). The cells were incubated at 37°C and 5% CO_2_. The experiment was conducted with extracts of all groups. Cells were seeded into 96-well cell culture plates at a density of 10^4^ cells/well and routinely incubated. After 24 h, the medium was removed and replaced with the prepared extracts, and culture was continued for another 24 h. Cell viability was tested using the MTT assay kit (Solarbio, Beijing, China) according to the manufacturer’s instructions. To display the cell viability more intuitively, the live/dead staining was carried out with Calcein-AM/PI Double Stain Kit (Solarbio, Beijing, China). Live cells were stained in green by Calcein-AM, whereas dead cells were stained red by PI. The staining results were captured under an inversion fluorescence microscope (BX51WI; Olympus, Tokyo, Japan).

#### 2.3.2 Cell Proliferation Activity

Cells were seeded into different materials with a density of 2 × 10^3^ /well. Cell proliferation was performed according to the CCK8 kit (CCK8, Dojindo, Japan) manufacturer’s protocol on 1, 3, and 5 days.

#### 2.3.3 Cell Adhesion Evaluation

For each group, 4 × 10^4^ cells were seeded onto the different samples, cultured for 24 h. The cell morphology was observed with SEM. In detail, the cells were rinsed with phosphate- buffered saline (PBS) and then fixed with 2.5% glutaraldehyde for 2 h. After the removal of the fixative, samples were rinsed with PBS again. Subsequently, dehydration was carried out with the gradient-ethanol method, with critical point drying in CO_2_ and gold coating. The cell morphology was visualized *via* SEM and photographed. To further validate the impact of the materials on cell morphology and cytoskeleton organization, the fluorescence staining of actin cytoskeleton was performed. In brief, cells were fixed with 4% paraformaldehyde (PFA) for 15–20 min, and then fixed cells were stained with FITC-phalloidin and DAPI (all Sigma-Aldrich, MO, United States) for cytoskeleton and cellular nuclei staining, respectively. Images were captured and recorded with the confocal laser scanning microscope (Fluo View FV-1000; Olympus, Tokyo, Japan).

#### 2.3.4 Cell Seeding Efficiency

To investigate the adhesive ability of cells on the materials over short time periods, the percent attachment was calculated after 4 h co-culturing time. MC3T3-E1 cells were seeded onto the materials at a density of 10^5^ cells/sample and incubated for 4 h. The medium was removed, and the cells were rinsed with PBS collected into the centrifuge tubes. Then, the residual cells on the materials were digested with trypsin (Solarbio, Beijing, China) and suspended in a suitable volume of culture medium, followed by counting with a cell-counting plate. The cell seeding efficiency was calculated as follows:

$$\text{η}=\frac{X}{X_{0}}$$

where η indicates the seeding efficiency, X the number of the residual cells, and X_0_ the initial number of cells seeded onto the samples.

### 2.4 Osteogenic Differentiation *in vitro*

MC3T3-E1 cells, which are pre-osteoblast derived from mouse calvaria, are used as model cells, being capable of differentiating to mature mineralizing osteoblasts. In this study, once the cells reached confluence, the culture medium was switched to osteogenesis inductive medium, composed of culture medium supplemented with 10 mM β-glycerophosphate, 10 nM dexamethasone, 50 μg/ml ascorbic acid (all Sigma-Aldrich, MO, United States).

#### 2.4.1 Alkaline Phosphatase Activity

MC3T3-E1 cells were seeded onto the different sample surfaces at a density of 2 × 10^4^ cells/well. The analysis of ALP activity and ALP staining was performed after osteogenically induction for 7 and 14 days. ALP was stained using an ALP staining kit (Beyotime, Shanghai, China) according to the manufacturers’ instructions. The intracellular ALP activity was also evaluated quantitatively. According to the manufacturers’ protocols, the BCA kit (Beyotime, Shanghai, China) was used to determine the total protein content and ALP activity was investigated by an ALP activity kit (Jiancheng, Nanjing, China).

#### 2.4.2 Alizarin Red s Staining

Detection of mineral deposition was performed using alizarin red staining and semi-quantification method. Cells were seeded onto the sample surfaces at a density of 2 × 10^4^ cells/well and cultured for 21 days with osteogenic medium. For ARS staining, cells were fixed in 4% paraformaldehyde and stained with 1% ARS (Solarbio, Beijing, China) for about 30 min. Images of the stained materials were taken by using a scanner (Perfection V600I; Epson, United States). To quantify calcium deposition, materials with cells were in treatment with 10% (v/v) cetylpyridinium chloride monohydrate (Aladdin, Shanghai, China) aqueous solution to extract the ARS dye, quantified at an OD of 562 nm (Gong L. et al., 2020).

### 2.5 Osteogenic Assay *in vivo*

#### 2.5.1 Rat Tibial Defect Model

Osteointegration of surfaces was evaluated by using the implant-plug model, creating the defective area in the proximal tibia of the rat. All surgical processes were approved by the Institutional Animal Care and Use Committee of Jilin University (IACUC No. 20210507-02). A total of 15 male Sprague–Dawley rats (n = 5) were in use (Weitong Lihua, Beijing, China). Animals were 12 weeks old and their mean body weight was 397 g. Animals were pre-anesthetized with isoflurane, and subsequently, an intraperitoneal injection of a urethane/acepromazine maleate mix was carried out to anesthetize the animal. Both hind limbs were prepared, and a 1 cm incision was made, with muscle released and the medial collateral ligament transected. The bone surfaces were exposed, and a 2.5 mm puncher (Integra Miltex, Plainsboro, NJ, United States) was used to generate holes in the bone tissues. Each implant was pressed into the hole, and it was made sure that the lip of the implant was flushed on the cortex. The incisions were sutured in a routine manner. Each animal was implanted one material in each leg, and experimental groups were randomized between animals and legs. All animals were euthanized after 8 weeks by carbon dioxide asphyxiation. Animal samples were fixed in PFA until further testing.

#### 2.5.2 Small Animal Microcomputed Tomography (μCT)

All samples were scanned with μCT prior to biomechanical testing and histologic examination. All the samples were examined using a μCT system (Bruker, Kontich, Belgium) to evaluate the amount of new-formed bones. Three-dimensional (3D) images of μCT were performed using the CTVox software, and bone regeneration was expressed as the bone volume/tissue volume (BV/TV) ratio and trabecular number (Tb. N) using Dataviewer and CTAn (all Brucker, Kontich, Belgium).

#### 2.5.3 Pull-Out Test

A biomechanical pull-out test was carried out to assess the osteointegration for each implant surface. All pullout tests were conducted using a universal testing machine (Instron 5500R, MA, United States). Each tibia was secured with a custom fixture, and the implant was linked to a 100 N load cell by passing a piano wire through the prepared hole. Pre-loaded samples (1.0 N) were placed to a constant tensile displacement rate of 0.1 mm/s. The tensile force is the maximum load reached before implant dislodgement or failure.

#### 2.5.4 Surface Patterns of Implants

Surface changes of implants were analyzed using SEM-EDX (energy-dispersive X-ray) system. SEM was used to observe and compare the implant surface morphology before and after implantation. Elemental analysis was performed using an EDX spectrometer for carbon, oxygen, calcium, and phosphorus, which represented osteogenesis-related elemental types.

#### 2.5.5 Histological and Immunohistochemical Analysis

After pull-out testing, the samples collected for histological analysis were fixed in 4% formalin and decalcified with 10% EDTA for 16 weeks until fully decalcified. After decalcification, all the specimens were paraffin-embedded and sectioned at 4 μm thickness and subjected to staining for histological evaluation. Histological analyses were conducted using hematoxylin and eosin (HE) staining and Masson’s trichrome (MTC) staining. Immunohistochemical staining for the bone morphogenetic protein-2 (BMP-2) and osteocalcin (OCN) of tissue sections was performed using a standard, indirect three-step immunoperoxidase technique.

### 2.6 Data Analysis

Statistical significance was determined by the independent-sample *t*-test for the comparison of two groups and one-way ANOVA for comparison of three or more groups. *p* < 0.05 was taken to indicate statistical significance, *p < 0.05 and **p < 0.01.

## 3 Results

### 3.1 Material Characterization

Po-PEEK materials obtained from the thermally induced phase separation technology are characterized by SEM, and the findings are reported in Figures 1B,C with two different magnifications, showing a network of well-connected pores with different dimensions. Figure 1D shows the differences and trends in the densities and porosity of different groups, indicating that the decline of densities is greater in the PK40 group (40% solid content), and that of PK50 (50% solid content) is rebounded somewhat. However, the densities of PK50 are increased with increasing degrees of compression, where PK50-10% or PK50-20% means that Po-PEEK with 50% solid content is compressed by 10% or 20%. The porosities as expected, show an opposite trend with the densities in the same groups. The mechanical strength tests of different groups are displayed in Figure 1E, and these results are consistent with the density results in the trend of change. Taken together, the PK50-20% group is the best outcome for density, porosity, and strength as the bone substitute materials. Therefore, PK50-20% was chosen for the subsequent experiments (pPEEK). For samples doped with nHA particles, a CT analysis of the composites (nHA-pPEEK) is presented in Figure 1F. As can be seen, HA nanoparticles embedded in pPEEK were clearly visible, showing the high-density shadows. The SEM image of nHA crystals is presented in Figure 1G, indicating that the synthetic nHA particles occur in the form of crystal clusters and the mean length of a single crystal is about 300 nm with a needle shape. The XRD result shows HA typical diffraction peaks and confirms the nucleation of HA in all samples (Figure 1H). Appropriate hydrophilic surfaces will be more conducive to initial cell attachment (Chen Y. et al., 2017). The hydrophilic–hydrophobic property of the different materials was characterized by water static contact angles (Figure 1I). The contact angle analysis shows 99° on the material surface of the control group while showing 50° and 21° on the material surfaces of the pPEEK and nHA-pPEEK groups. It is generally believed that contact angles greater than 90° indicate hydrophobic properties, while smaller than 45° denotes clearly hydrophilic ones (Peters, 2017). Thus, as far as hydrophilic properties, statistically significant difference is observed among the control groups (PEEK and pPEEK) and experimental groups (nHA-pPEEK) (*p* < 0.01). Interestingly, the hydrophilic ability of pPEEK is better than PEEK (*p* < 0.05), which means that the porous structure may also improve the hydrophobicity of the original biomaterial.

### 3.2 Cellular Compatibility

**FIGURE 2:**
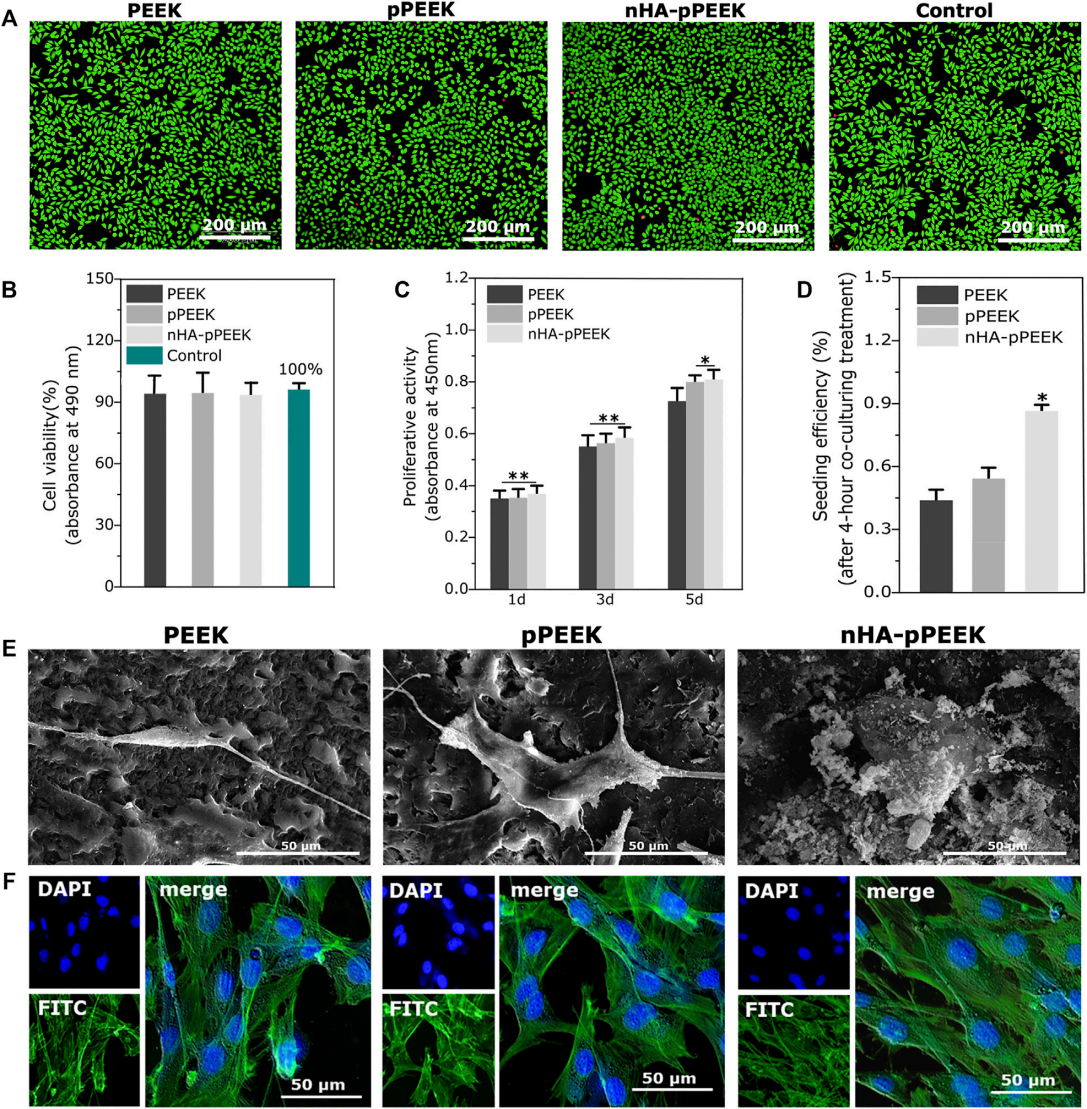
Biocompatibility of prepared materials. **(A)** Live/dead cell staining for evaluation of different extracts from all groups. **(B)** Cell cytotoxicity quantitative measurement by the MTT assay. **(C)** Analysis of cell proliferation on different surfaces of all groups. **(D)** Seeding efficiency for MC3T3-E1 cells on the different materials. **(E)** Cell morphology in SEM of MC3T3-E1 osteoblast cells after co-culture with different samples. **(F)** Fluorescent staining of cells with FITC-phalloidin (actin cytoskeleton, green) and DAPI (nucleus, blue).

The conditions of the cultures contacted with extracts from the materials (indirect methods) are assessed using image analysis and quantitative analysis. Results are presented in Figures 2A,B), and all groups do not show any toxicity, thus showing that the synthetic materials are cell compatible completely. To investigate possible influences of the material surface on the proliferation of the cells, CCK8 assessment is taken, and the results are shown in Figure 2C. The cell proliferation in each group increases based on the culturing time, and all groups show higher values than the frontier group (*p* < 0.01). On day 5, pPEEK and nHA-pPEEK groups show higher values than the PEEK control group (*p* < 0.05), which means that a porous structure may promote the ability of cell proliferation, but nHA does not show apparent superior performance. Cell seeding efficiency is a cell adherence assay to evaluate the effect of materials on cell adhesion. From the results (Figure 2D), nHA-pPEEK can stimulate the adhesion of MC3T3-E1 cells compared with other groups (*p* < 0.05). The morphology of MC3T3-E1 cells adhered on the surfaces of materials is observed under SEM (Figure 2E), revealing that nHA-pPEEK can promote the expansion of cells apparently from the difference in cellular area. Cell morphology and spreading are closely related to cytoskeletal structure, so cell fluorescent staining is performed. Results are displayed in Figure 2F, where nuclear staining is blue and cytoskeletal staining is green. Compared to PEEK and pPEEK groups, there are a greater number of cells and larger amounts of cytoskeleton fibers of each cell in the nHA-pPEEK group. The cytoskeleton is the key component that regulates cell migration and differentiation, and some studies believe that osteogenic gene activation is mediated by cytoskeletal change (Wei et al., 2020).

### 3.3 *In Vitro* Osteogenic Differentiation Assays

**FIGURE 3:**
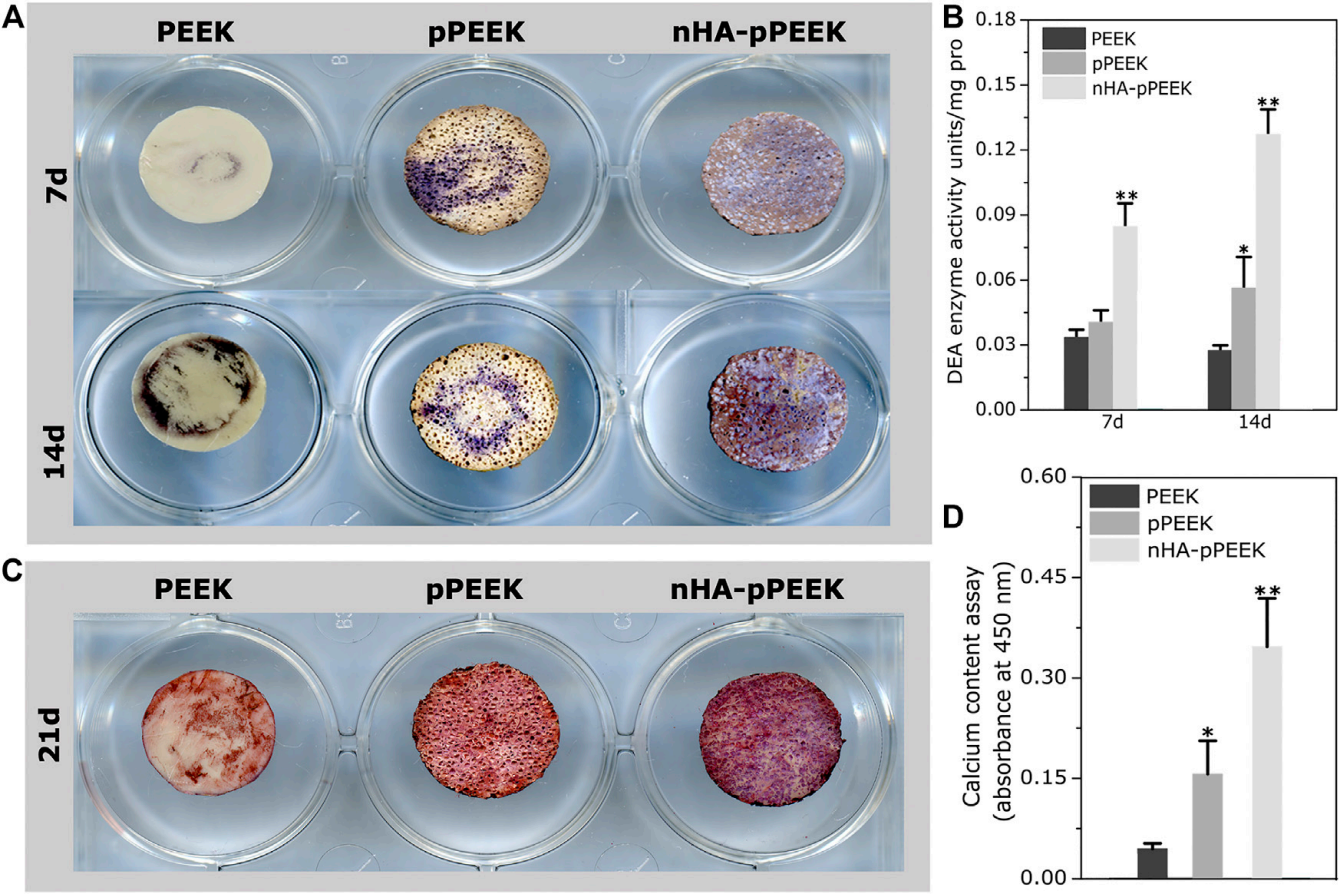
Osteogenic differentiation of MC3T3-E1 cells. **(A)** ALP staining assays of cells on the different materials after 7 and 14 days co-culture time. **(B)** ALP activity in cell lysates collected from cells co-cultured with different materials. **(C)** ARS staining of cells on the different materials. **(D)** Quantification of ARS staining in **(C)**.

We have analyzed the osteogenic potential of the synthetic materials *in vitro*. ALP activity is a marker of early-stage osteogenesis (Chen E. E. M. et al., 2017). The samples show different degrees of ALP staining (Figure 3A), where the results in day 14 are greater than day 7 overall. At each specific time point, the nHA-pPEEK group shows deepest staining, indicating that ALP activity is highest, followed in decreasing order by the pPEEK and PEEK groups. To investigate whether the differences among the groups are highly significant, ALP activity quantitative assessment is performed (Figure 3B). In agreement with staining results, there are statistically important differences in ALP activity between the nHA-PEEK and other groups (*p* < 0.01). Notably, the ALP activity in the pPEEK group on day 14 is higher than PEEK groups (*p* < 0.05), which means that pPEEK has a certain degree of osteoinductive abilities. Mineralization is considered an *in vitro* endpoint of osteogenic differentiation (Lin et al., 2017). Thus, ARS staining is performed on day 21 and results are presented in Figure 3C. The red color is deeper and deeper in order of PEEK, pPEEK, and nHA-pPEEK, indicating that the content of calcium is more and more. The result of quantification of ARS is consistent with the result of ARS staining (Figure 3D), where the nHA-pPEEK group has a higher content of calcium than other groups (*p* < 0.01), and pPEEK is also slightly elevated compared to PEEK (*p* < 0.05).

### 3.4 *In Vivo* Bone Integration

**FIGURE 4:**
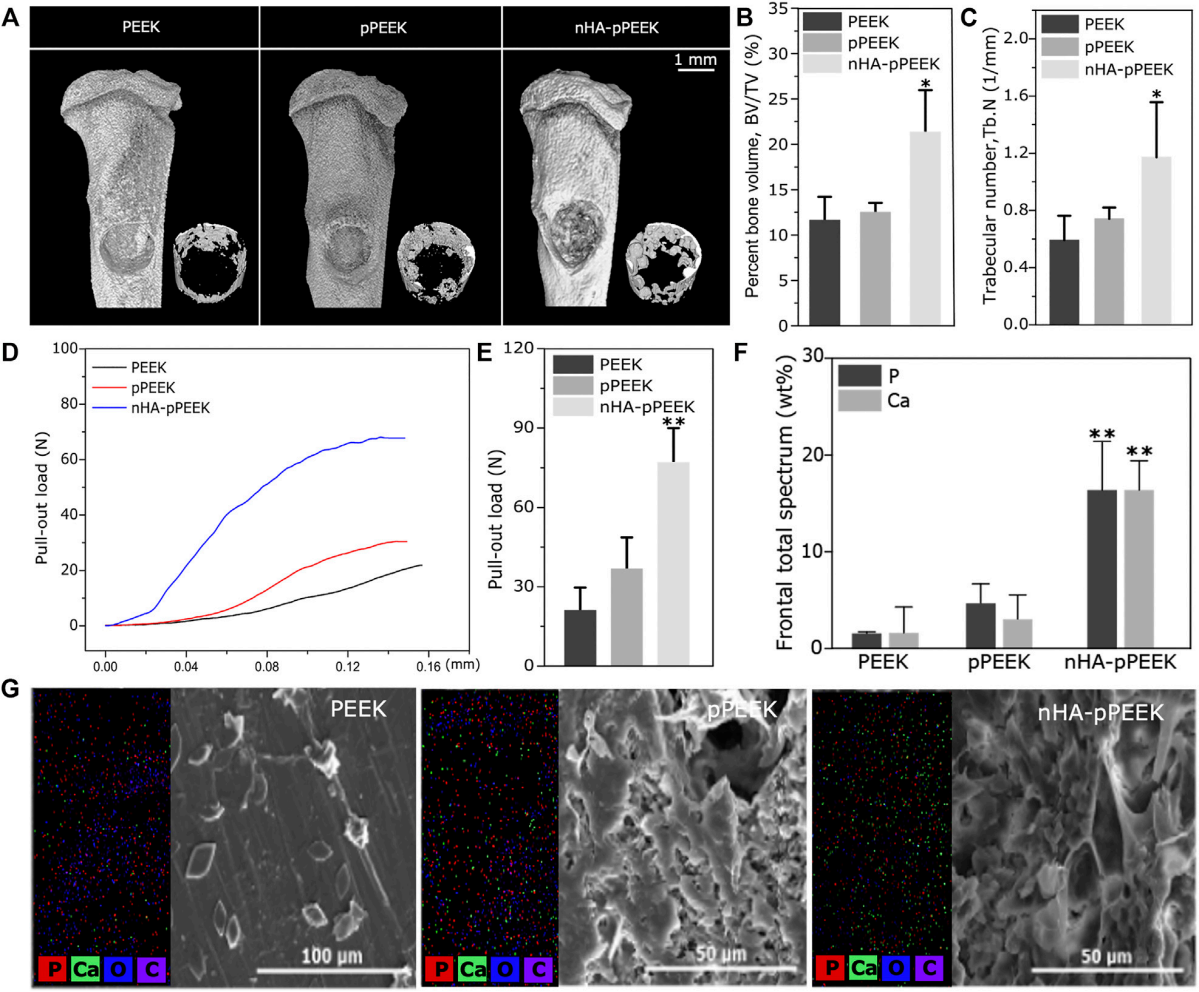
*In vivo* analysis on osseointegration. **(A)** Three-dimensional images obtained from μCT reconstruction. Overall pattern of tibia (upper); new bone in the defect part (lower). **(B)** BV/TV, and **(C)** Tb. N of the defective aera calculated using accessory software. (D) Pull-out strength of the implanted materials in all groups, having different effects on osseointegration. **(E)** The maximum values of pull-out load taken for quantitative analysis. **(F)** Quantitative analyses of phosphorus and calcium. **(G)** The surface composition of each sample analysis by SEM-EDX system, including SEM images and distribution maps of the elements of carbon, oxygen, phosphorus, and calcium.

**FIGURE 5:**
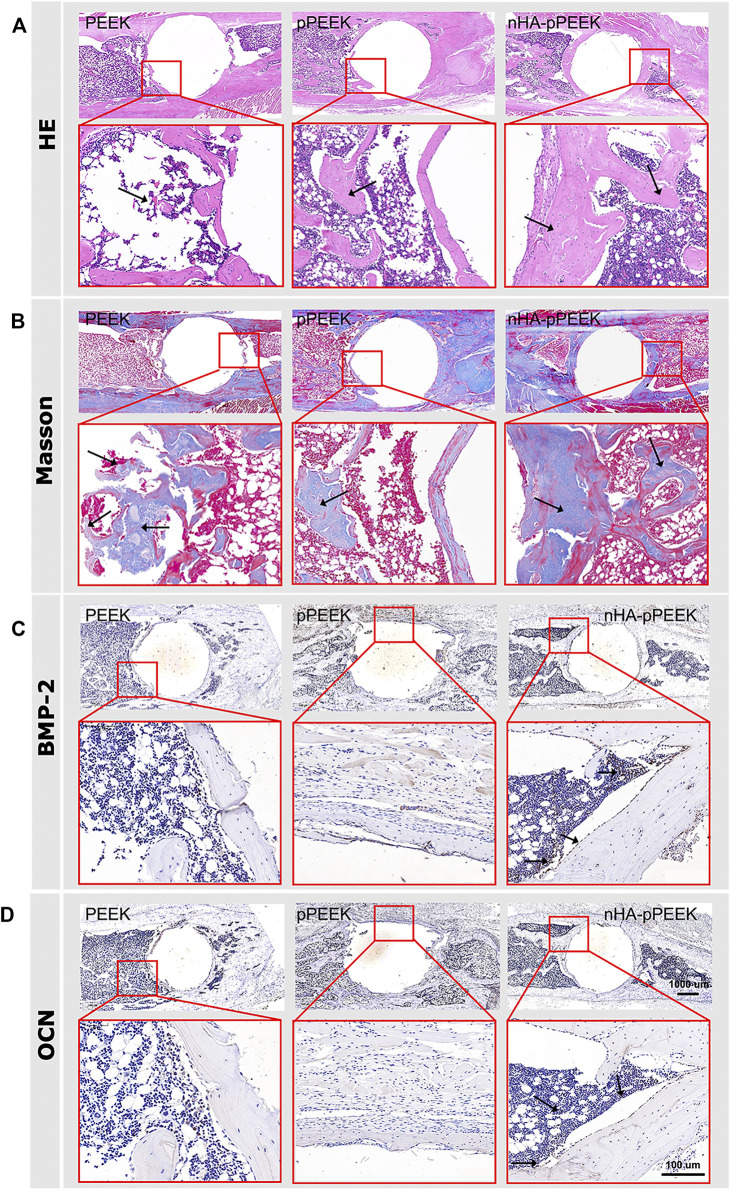
Histological analysis. **(A)** Representative displays of hematoxylin–eosin (HE) staining of tibia tissue sections, revealing that nHA-pPEEK composites markedly promoted new bone formation (black arrows). **(B)** Results of MTC staining for the collagenous fiber distinguished as blue (black arrows), showing that the experimental group had a large number of blue collagen fibrils and fibrous tissue. **(C)** Results of BMP-2 protein immunolocalization *via* immunohistochemistry staining to make a successful differential diagnosis (black arrows). **(D)** OCN immunolocalization *via* immunohistochemistry staining to make a successful differential diagnosis (black arrows).

To demonstrate implant functionality, the osseointegration of implants placed around rat tibias is analyzed. Eight weeks after the implantation, materials with surrounding tissues are harvested for analysis. The μCT scanning-reconstructed images for different groups are shown in Figure 4A. From the results, bone density and the amount of new bone volume in nHA-pPEEK are apparently higher than the PEEK and pPEEK groups, which indicates that the osteoinductivity and osteoconductivity of nHA-pPEEK composites are greater than others. Results of bone parameters, including BV/TV (Figure 4B) and Tb. N (Figure 4C), are consistent with reconstructed images, showing that nHA-pPEEK has the best performance in osteogenesis (*p* < 0.05). The biomechanical pull-out test is utilized to evaluate the quality of osseointegration of the implants, and the results (Figures 4D,E) show that there are significant differences observed among materials (*p* < 0.01), which illustrates that nHA-pPEEK has the ability to enhance the osteogenic capacity of biomaterials. After pull-out, SEM is employed to characterize the morphology of the surfaces on the implants. Results show that new bone tissues deposit on the surface of the materials in each group and intensity differs among the groups (Figure 4G). Quantification indicates statistical significance (Figure 4F, *p* < 0.01), further confirming the osteogenic capacity of the nHA-pPEEK implants. According to the results of HE and Masson staining (Figures 5A,B) 8 weeks after bone graft in the tibiae of rats, the osteogenic area can be clearly seen in the nHA-pPEEK group, which shows cell aggregation at the osteogenic front of the newly formed bone and where the area presents collagen fiber bundles of high content. Figures 5C,D shows that the osteogenic differentiation of cells was observed by positive immunohistochemical staining for BMP-2 and OCN and the results confirm that the nHA-pPEEK materials can effectively promote stem cells to differentiate to the bone cells compared to other groups.

## 4 Discussion

HA-based biomaterials are widely used as bone substitute material because its osteoinductive activity is generally recognized, because biosafety is a prerequisite for bone- substitute *in vivo* applications (Tang et al., 2017). To date, there are many ways in which HA participates in the PEEK materials, as described before, but with a variety of weaknesses consistently present. In this paper, a new technique based on thermally induced phase separation method and hydrothermal synthesis technology is used for producing bioactive nHA-pPEEK composites. The technique is also an efficient method for fabricating Po-PEEK with changeable porosity. The nHA-pPEEK biocomposites produced exhibit good biocompatibility and cell attachment, while the incorporation of nHA into PEEK does not result in the reduction of mechanical strength because the HA particles are attached on the surface rather than embedded in PEEK substrate. SEM and XRD results confirm the formation of crystalline nHA on the porous pPEEK materials after the hydrothermal method. Upon the addition of nHA biomolecules, nHA-pPEEK composites’ hydrophilic properties are improved compared with other groups. The surface hydrophilicity will promote the attachment of many different cells, and cell spreading, proliferation, and differentiation have also been related to it in addition to initial cellular attachment (Pan et al., 2015; Pajoumshariati et al., 2018).

In the biomedical field, cell viability is often used to determine the biocompatibility of biomaterials. According to ISO 10993-5, cell viability results can be interpreted as following: >80% no cytotoxicity, 80%–60% weak cytotoxicity, 60%–40% moderate cytotoxicity, and <40% strong cytotoxicity (Ciorita et al., 2020). The results show that all fabricated materials are compatible with MC3T3-E1 cells with no cytotoxicity. Cell proliferation is another of the cell activities required for tissue regeneration (Del Castillo-Santaella et al., 2019). The cell proliferation rate of cells cultured on the material surfaces changes at different time points, and the results in the pPEEK and nHA-pPEEK groups are superior to that in the control group on day 5, which means that porous structure can promote cell proliferation and nHA particles have no proliferation-promoting effect significantly despite the higher levels of cell viability (*p* > 0.05). This is probably because the porous structures allow for the optimal interaction of the materials with the cells, oxygen and other nutrients are delivered to nourish the attached and embedded cells (Tong et al., 2016). The material properties can influence cellular reaction when cells attach the surfaces, and material–cell interactions are further evaluated by cell adhesion tests (Cui et al., 2017). Cell seeding efficiency on the surfaces of synthetic materials in a short period of time correlates with almost surface property changes, and nHA particles occupy a preponderant place in the cell attachment. Next, the morphologies of cell spreading within 3D matrix are obtained using SEM after co-culturing with different groups for 24 h. The results show that cells have a more homogenous spreading distribution in the nHA-pPEEK group, which is possibly associated with cell differentiations. Hence, the better performance of nHA-modified samples in improving cell differentiation is probably associated to the functions and bioactivity of nHA (Jiang et al., 2020). Cell spreading is often accompanied by changes in the structure and composition of the cytoskeleton (Ma et al., 2016), and the beneficial outcome of cytoskeleton staining further confirms the effect of nHA and porous structure on MC3T3-E1 cells. The cell-spreading area and the cell numbers can be helpful data for cell evaluation on materials.

During early stages of cell differentiation, osteoblasts synthesize ALP and other related markers, which ultimately leads to the extracellular matrix calcification, where the degree of osteoblastic differentiation is evaluated by the expression of the aforementioned osteoblast markers (Zhang et al., 2017). The expression of ALP is monitored using ALP staining and ALP activity assay. Results show that a significantly greater ALP activity is observed in the nHA-pPEEK group compared with other groups at any point in time. Interestingly, cells in the pPEEK group have a higher level than the PEEK groups in the ALP activity quantification experiment, which is possibly related to the morphology of porous structure (Feng et al., 2020). The degree of the extracellular matrix mineralization is determined using ARS staining and the semi-quantification of cultured osteoblasts to detect bone nodules. Results indicate that the number of calcium nodules is increased obviously in the nHA-pPEEK group, and a mild increase is observed in the pPEEK group, which is consistent with the ALP quantification results above. In the osteogenic experiments, nHA-pPEEK composites show better osteogenic ability with enhanced ALP activity and more calcium deposition. Lastly, the current results are obtained from *in vitro* experiments and need to be validated *in vivo*.

In this study, the effect of synthesized composite materials on osteointegration is investigated in a well-established rat tibial bone defect model. At the end of the eighth week, rats are sacrificed, and tibias are detected by μCT, and the data reveal that the amount of new bone formation on the surfaces of the nHA-pPEEK group is significantly higher than other groups, showing a high amount of newly formed bone in the defect areas. A quantitative analysis involving BV/TV and Tb. N further tests this idea. The osteointegration of the newly formed bone is examined by the pull-out test, and the results are expressed as the maximum pull-out force, which is consistently higher for the nHA-pPEEK than for the pPEEK, and pure PEEK groups. This could be partially explained by biomimetic mineralization (Zhao et al., 2020). Porous nHA-pPEEK composites mimic the structure of natural bones in the sense that nHA acts as the bone salt and pPEEK the porous structure to cancellous bone. The biomechanical strength of bone–implant integration is measured by the pull-out test, and the surfaces of the extracted materials are examined by SEM-EDX for the analysis of new bone formation. The present experimental results show that the pull-out force is the maximum in the nHA-pPEEK group, hindered by the newly formed coupled bone. SEM images show that the nHA-pPEEK composites appear to have a rough surface morphology. The measurements of calcium and phosphorus on the surfaces performed with EDX identify these elements in all treatment groups, but the nHA-pPEEK group has the greatest amount compared to others and based on the results of the quantitative analysis. The HE histological staining is used to demonstrate different tissue structures, and the newly formed bulk bone tissues are displayed, where the collagenous regions contain a lacuna structure that is surrounded with osteoid. The results of Masson staining are expected, which show that the nHA-pPEEK group has the most blue staining area of collagen fibrils. BMP-2 and OCN are the respective mid-later stage markers in the osteogenic differentiation process of stem cells (Shuang et al., 2016; Shalumon et al., 2018; Dashtimoghadam et al., 2020), and so the evaluation is necessary to assess. There is significantly greater positive OCN or BMP-2 staining in the nHA-pPEEK group compared with other two groups, which illustrates the successful osteogenic differentiation of MC3T3 cells, further proving the excellent osteoinductive performance of the nHA-pPEEK composites. The *in vivo* experiment further indicates that nHA-pPEEK induces more new bone formation and tighter bone bonding.

## 5 Conclusion

In this study, an nHA-pPEEK was developed and its potential to successfully promote bone integration *in vivo* was demonstrated. The nHA-pPEEK composites were fabricated by thermally induced phase separation method and hydrothermal reaction technology. In the *in vitro* experiments, the composites displayed good osteogenesis and biocompatibility. After 8 weeks of implantation, significantly higher new bone formation within the defective area as well as at the implant–bone interface was achieved in the nHA-pPEEK group compared to that of the PEEK and pPEEK groups. The results showed that the nHA-pPEEK biomaterials can be a promising bone substitute for the treatment of bone defects.

## Data Availability

The original contributions presented in the study are included in the article/Supplementary Materials, further inquiries can be directed to the corresponding author.
